# Biocompatibility of titanium from the viewpoint of its surface

**DOI:** 10.1080/14686996.2022.2106156

**Published:** 2022-08-15

**Authors:** Takao Hanawa

**Affiliations:** a Institute of Biomaterials and Bioengineering, Tokyo Medical and Dental University, Tokyo, Japan; b Center for Advanced Medical Engineering Research and Development, Kobe University, Kobe, Japan; c Division of Materials and Manufacturing Science, Graduate School of Engineering, Osaka University, Osaka, Japan

**Keywords:** Titanium, passive film, corrosion resistance, surface electric charge, electrostatic force, band gap energy, biocompatibility

## Abstract

Among metals, Ti and majority of its alloys exhibit excellent biocompatibility or tissue compatibility. Although their high corrosion resistance is a factor in the biocompatibility of Ti and Ti alloys, it is clear that other factors exist. In this review, the corrosion resistance and passive film of Ti are compared to those of other metallic biomaterials, and their band gap energies, *E_g_s*, are compared to discuss the role of *E_g_* in the reactivity with living tissues. From the perspective of the material’s surface, it is possible to explain the excellent biocompatibility of Ti by considering the following factors: Ti ions are immediately stabilized not to show toxicity if it is released to body fluids; good balance of positive and negative charges by the dissociation of surface hydroxyl groups on the passive film; low electrostatic force of the passive film inducing a natural adsorption of proteins maintaining their natural conformation; strong property as *n*-type semiconductor; lower band gap energy of the passive film on Ti generating optimal reactivity; and calcium phosphate formation is caused by this reactivity. The results suggest that due to the passive oxide film, the optimal balance between high corrosion resistance and appropriate reactivity of Ti is the predominate solution for the excellent biocompatibility of Ti.

## Graphical abstract

**Figure:**
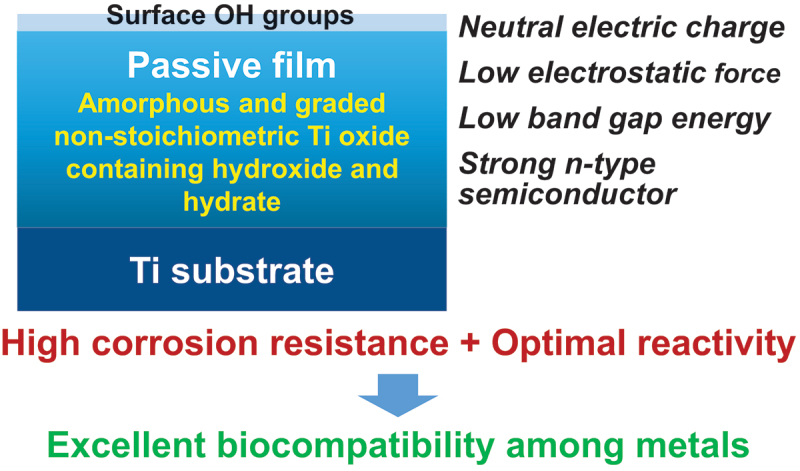

## 1. Introduction

**Figure 1.**
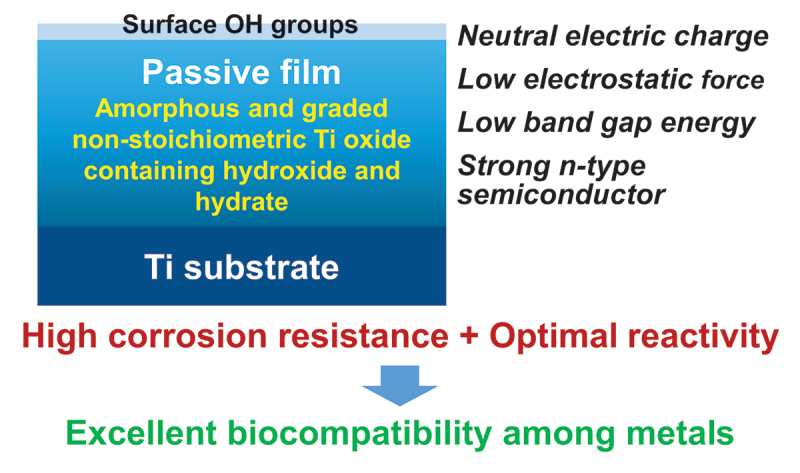
Illustration of items reviewed and explained in this review.

Commercially pure titanium (CP Ti) and majority of Ti alloys exhibit excellent biocompatibility or tissue compatibility, as demonstrated by a number of studies and clinical findings [1]. However, the dominant mechanism of the biocompatibility of Ti (CP Ti and Ti alloys) is not known, although their high corrosion resistance is a factor. Biological approaches, including interfacial observation between Ti and the surrounding tissue, gene expression of cells on Ti, protein adsorption to Ti, peptide sequence of adsorbed proteins to Ti, bone formation on Ti, soft tissue adhesion to Ti, and bacterial adhesion to Ti, have been utilized to elucidate the mechanism of biocompatibility [1,2]. Ti and zirconia (ZrO_2_) ceramics are compared as dental implant materials from the perspectives of mechanical property, bone formation, soft tissue adhesion, and antibacterial property [3]. However, Ti is typically classified as a bioinert material because its bioactivity is significantly less than that of bioactive ceramics [4]. Therefore, the focus of Ti research as a biomaterial has shifted to the improvement of biocompatibility, *i.e*., the development of surface treatment techniques. To demonstrate the efficacy of surface treatments, the biocompatibility of Ti as a control is typically disregarded: Ti is bioinert. In any case, the principle of Ti’s biocompatibility cannot be determined solely through biological approaches, as the biocompatibility’s origin lies in the material, particularly its surface. In this review, the corrosion resistance and passive film of Ti are compared to those of other metallic biomaterials, and their band gap energies, *E*_g_s, are compared to discuss the role of *E*_g_ in the reactivity with living tissues. Figure 1 depicts a summary of the topics covered in this evaluation. Terminology in this review, ‘reactivity’ includes not only photocatalysis and corrosion but also protein adsorption, calcium phosphate formation, and other reactions occurring at the surfaces of materials.

## 2. Biocompatibility of Ti

**Figure 2.**
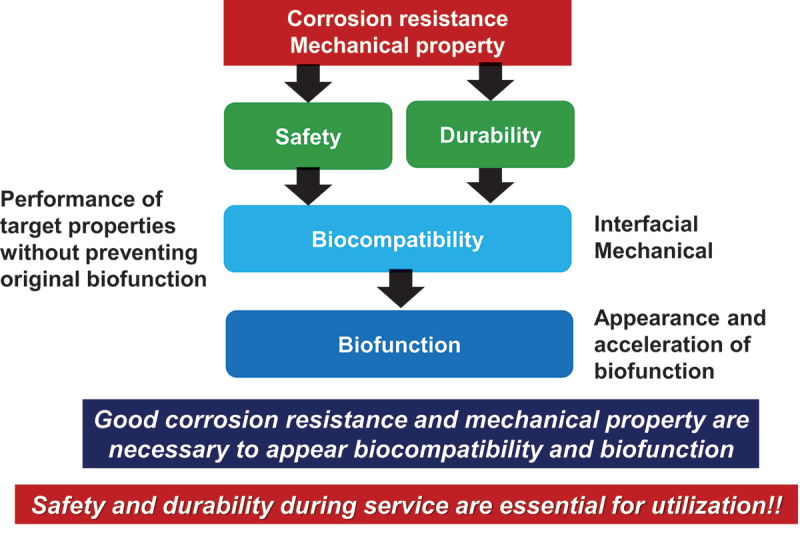
Corrosion resistance and mechanical property as necessary condition for the biocompatibility and biofunction.

Biocompatibility is defined as ‘the ability of a material to perform in a specific application with an appropriate host response [5]’. The biocompatibility of a material is determined by initial and continuous reactions between the material and host body, such as molecule adsorption, protein adsorption, cell adhesion, macrophage activation, tissue formation, bacterial adhesion, and inflammation, etc. In addition, a temporal and spatial hierarchy governs the reaction [2]. When dissolved metal ions from metals in the human body react with biomolecules or cells, disrupting their functions, metal ions have been identified as being toxic to living organisms. To avoid this toxicity, metals used for medical implants must have a high corrosion resistance in the presence of living tissue. Consequently, corrosion resistance is a necessary condition for biocompatibility, as illustrated in Figure 2, but it is not a sufficient condition, as described below.

‘Osseointegration’ is a property unique to Ti among metals [6]. Osseointegration is defined as follows. It is the ‘formation of a direct interface between an implant and bone, with no soft tissue intervening. There is no connective tissue, cartilage, or ligament fibers between the bone and implant surface. Microscopically, the direct contact between bone and implant surface can be confirmed [6]’. This concept, osseointegration, in dental implants generated and accelerated studies on the reaction between hard tissues and Ti, followed by surface treatment studies. Studies on: evaluation of osteoblast calcification; histological evaluation, such as bone formation, bone-contacting rate, and bone bonding strength; and clinical results have demonstrated Ti’s excellent compatibility with hard tissues. Important determinants of hard-tissue compatibility are the adhesion and proliferation of osteogenic cells as a result of the surface morphology (roughness), wettability, and other characteristics. Ti-bone interface reaction has been characterized to demonstrate the significance of surface morphology and wettability for osseointegration [7–9]. Numerous studies on the compatibility of Ti with hard tissues have been conducted, and detailed information is available in the literature [1,2]. In orthopedics, bone screws and bone nails made of Ti alloys typically form calluses and assimilate into bone tissue after long-term implantation, causing the bone to refracture during retrieval [10]. This is due to the fact that Ti alloys are compatible with hard tissues.

## 3. Corrosion behavior of metallic biomaterials

### 3.1. Corrosion resistance of Ti and Ti alloys

**Figure 3.**
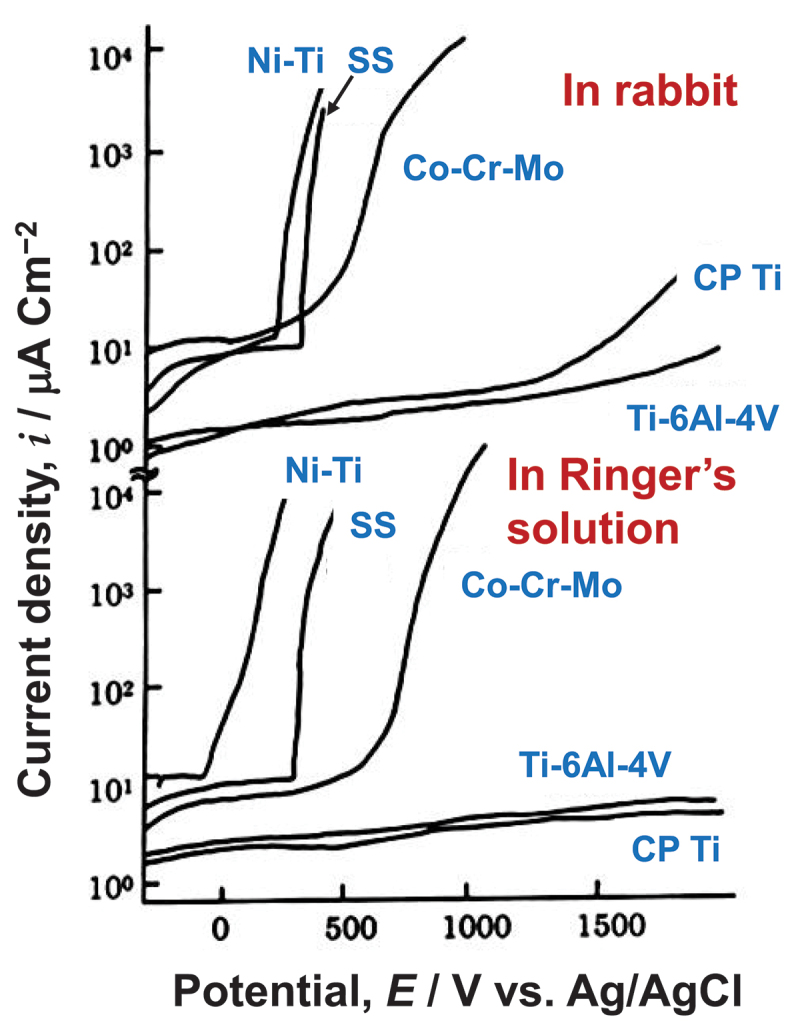
Anodic polarization curves of CP Ti, Ti−6Al−4V alloy, Co−Cr−Mo alloy, type 316L stainless steel (SS), and pure Ni in rabbit and Ringer’s. Reproduced by permission from [13], copyright [1989, Elsevier].

Numerous studies confirm the Ti’s superior corrosion resistance in biological environments. CP Ti, Ti–6 aluminum (Al)–4 vanadium (V) alloy, nickel (Ni)–Ti alloy, cobalt (Co)–nickel (Ni)–chromium (Cr)–molybdenum (Mo) alloy, Co–Cr–Mo alloy, type 316 L stainless steel, and pure Ni exhibit the strongest passivation in this order in Hanks’ physiological solution at 37°C and 7.4 pH [11]. Afterwards anodic polarization measurements of several orthopedic implant metals and alloys, including type 316 L stainless steel, Co–Cr–Mo alloy (ASTM F-75), Ni–Ti alloy, pure Ni, CP Ti, and Ti–6Al–4 V alloy, are performed in Ringer’s solution with and without 1% bovine serum albumin [12] and in Ringer’s solution and rabbit [13], which demonstrates the excellent corrosion resistance of CP Ti and Ti, as shown in Figure 3. Recent studies [14,15] have produced comparable results. The corrosion behaviors of the aforementioned materials have been thoroughly reviewed [16]. Both CP Ti and Ti-6Al-4 V alloy demonstrate much lower passive currents and higher breakdown potentials without pitting in vitro and in vivo.

### 3.2. Ti element released to the surrounding tissues

Despite the high corrosion resistance of Ti, since more than three decades, numerous studies [17–22] have demonstrated that despite the absence of abrasion, a significantly greater quantity of Ti elements is detected in the surrounding tissues when Ti materials are implanted.

The effect of amino acids and proteins on the solubility of metals is examined [23,24]. Possible Ti ion dissolution mechanisms are examined from the perspective of the isoelectric point and the electric charge of proteins contained in body fluids [25]. Mo, copper (Cu), Co, and Ni ions are released when pure metal powders are immersed in saline, with or without serum albumin or fibrinogen, but Ti ions are not released and are unaffected by the presence of proteins [26]. In the case of Ti-6Al-4 V, Ti and Al ions are released in Hanks’ solution containing 2% EDTA, whereas Ti, Al, and V ions are released in Hanks’ solution containing 0.05-M sodium citrate [27]. Ni – Ti alloy initially releases more Ni than stainless steel immersed in a medium containing osteoblasts or fibrinogen, but the amount released decreases after two days [22]. As a result, metal ions are released in rabbits in the absence of wear and are detected in the rabbit’s tissues, serum, and urine. Fretting corrosion depends on (the charge of) proteins; in the presence of proteins, Ni preferential dissolution increases [25]. Biomolecules may account for the release of metal ions. Although the mechanism for the accelerated release of metal ions in the presence of amino acids and proteins has not been elucidated, an imbalance between partial dissolution and re-precipitation in the passive film may accelerate the release of ions. Therefore, repassivation of a metal influences the release of ions from the metal. During the repassivation of Ti in aqueous solutions, inorganic ions, and proteins accelerated the repassivation of Ti, whereas certain amino acids slowed it [28].

Immunological reactions and the adhesion of macrophages (Mϕ) to the surface of an implanted material identify it as a foreign body [29]. Mϕ generates active oxygen species, H_2_O_2_, which has a much longer lifetime and higher permeability against cell membrane than O^2^^‒:^ H_2_O_2_ reaches the surface to which Mϕ has adhered, and the Ti surface is hyperoxidized by H_2_O_2_ [30,31], which may result in the release of Ti ions. H_2_O_2_ reacts with the passive film on Ti according to the following equation [31]: Ti^4+^ + H_2_O_2_ → Ti^5+^ + OH^‒^ + OH*, where * represents radical. Dissolution of Ti with active oxygen generated by Mϕ has been elucidated adequately [32]. On the other hand, surgical handling during implantation and wear and/or fretting were the leading causes of Ti release [33].

**Figure 4.**
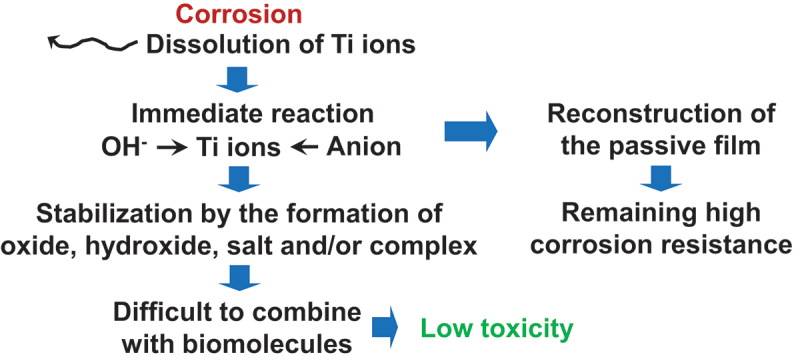
Mechanism remaining low toxicity even though Ti is corroded.

Regardless, despite the detection of Ti element in the surrounding tissues, the toxicity of Ti materials has hardly manifested. In the majority of instances involving the detection of released Ti elements, the chemical states of these elements are obscure. As depicted in Figure 4, dissolved Ti ions combine immediately with hydroxide ions and anions to stabilize the Ti element in the human body and are utilized for the reconstruction of the passive film. Therefore, the possibility of Ti surviving as ionic states and combining with biomolecules is extremely low. Consequently, Ti exhibits low toxicity.

### 3.3. Stainless steel

Ti alloys have replaced the majority of stainless steels used for the stems of artificial hip joints and bone fixators due to their lower corrosion resistance. However, stainless steel is still utilized for retrievable internal bone fixators and sternal and bone fixation wires due to its superior torsion property and elongation to fracture. Stainless steels are also utilized for medical and surgical instruments and equipment. For implant materials, type 316 L austenitic stainless steel always used. Adding 2.0–3.0 mass% of Mo, increasing Ni from 8.0–10.0 mass% to 12.0–15.0 mass%, and decreasing carbon (C) to less than 0.030% increases its corrosion resistance [34]. The presence of Mo reduces both the number of nucleations and the size of metastable pits. This is due to the strengthening of bonds in the passive film and the elimination of active pitting sites caused by the formation of molybdates or molybdenum oxyhyroxides [35].

In biological environments, type 316 L stainless steel typically exhibits pitting corrosion due to anodic polarization, as shown in Figure 3. Severe crevice corrosion of spinal rods is observed in the human body [36]. In addition, severe corrosion pitting was observed on sternal wires implanted for over 30 years [37]. Consequently, the corrosion resistance of type 316 L stainless steel is considerably less than that of Ti and Ti alloys.

### 3.4. Co–Cr alloys

Co–Cr alloys show excellent mechanical properties, castability, corrosion resistance, and wear resistance [34]. The absence of crevice and pitting corrosion in Co–Cr alloys is confirmed by anodic polarization in simulated bioliquids [38], and the Co–Cr alloys have a high localized corrosion resistance that is independent of a small change in composition [39]. Their corrosion resistance is superior to that of stainless steel. Their wear resistance is superior to that of stainless steel, CP Ti, and Ti alloys.

In the field of orthopedics, a cast Co–Cr–Mo alloy known as ‘Vitallium’ (ASTM F75) is utilized for artificial knee joints and artificial hip joints, in particular, the heads. ASTM F79 Co–Cr–Mo alloy is produced by refining the grain of F75 using hot rolling. The F99 alloy has twice the tensile strength and yield strength of the F75 alloy. To improve the workability of Co–Cr–Mo alloy, ASTM F90 as ‘HS25’ or ‘L-605’, used for orthopedic wire, is developed by reducing the C content and adding W and Ni, resulting in a workability of 44% and a strength after working that is more than double that of F75 alloy. ASTM F562 Co–Ni–Cr–Mo alloy as ‘MP35N’ is used in cardiovascular surgery for stents with superior strength, elasticity, and corrosion resistance, and tensile strength exceeding 1600 MPa. This alloy, along with the ASTM F90 alloy, has a high elastic modulus, which makes it advantageous for use as a stent. On the other hand, ASTM F1058 Co–Cr–Ni–Mo–iron (Fe) alloy, also known as ‘Elgiloy’, is utilized for artificial heart springs and aneurysm clips. Co–Cr–Mo alloy is utilized in dentistry for removable partial dentures with clasp, crowns, and bridges [34].

However, Co–Cr alloys can experience metallosis due to the Co release. A 56-year-old female with metal neuropathy and a Co–Cr alloy hip prosthesis developed metallosis. Co and Cr levels in her blood decreased after exchange arthroplasty, and her symptoms improved. Elements containing Co or Cr can cause axonopathy [40]. Cases with pseudotumors typically indicate poorly functioning implants and have significantly higher median metal ion concentrations: median Co levels were found to range from 6.9 to 29.7 μg/L [41]. Co–Cr alloy implant particles were associated with persistent, dose-dependent peri-spine inflammation [42].

**Figure 5.**
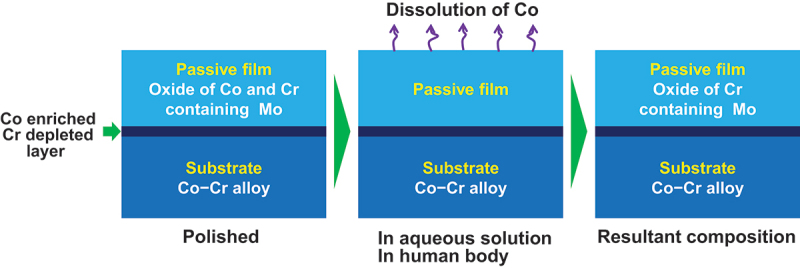
Co ions are released from initial passive film in aqueous solutions or the human body and the resultant passive film is Cr oxide containing small amount of Mo.

The primary cause of the aforementioned metallosis is the release of Co ion. Co ions released from the passive film on the alloys [43–47]. During releasing Co ions, Cr, Mo, tungsten (W), and Ni are enriched in the passive film, whereas Co is depleted, in accordance with their oxidation and reduction potentials. Figure 5 illustrates this occurrence. Therefore, under wear conditions, Co ions are released repeatedly [48,49], and the amount of Co ions released may be substantial. Therefore, the above clinically observed metallosis is due to the release of Co ions following implantation.

### 3.5. Ni–Ti alloy

Ni–Ti alloys composed of equal amounts of Ni and Ti (49–51 mol%Ni) exhibit exceptional mechanical properties, including shape memory, superelasticity, and damping. The Ni–Ti alloy is utilized for guide wires, stents, orthodontic arch wires, and endodontic files due to its exceptional properties [34].

As stent grafts, Ni–Ti alloys exhibit severe pitting and crevice corrosion. The observed corrosion defects of pitting and irregular shape are precursors to material failure. They weaken the thin wire, resulting in stress cracks and eventual fracture of the stent wire when subjected to circulation pulses [50]. Although the materials chosen for the construction of endovascular grafts appear prudent, the assembly of these biomaterials into various interconnected structures within the device requires further development [51].

### 3.6. Summary of corrosion

Metals with the best corrosion resistance in biological environments are Ti and its alloys. Co–Cr alloys also exhibit good corrosion resistance in the absence of pitting and crevice corrosion; however, the alloys release Co ions in aqueous solution to stabilize the passive film, so Co ions are repeatedly released under wear conditions. Type 316 L stainless steel and Ni–Ti alloy exhibit pitting and crevice corrosion on occasion. Therefore, Ti has the highest corrosion resistance among metals.

## 4. Relationship between corrosion resistance and biocompatibility in Ti

The corrosion resistance of Ti is one of the reasons for its excellent biocompatibility; however, corrosion resistance is not a sufficient condition for biocompatibility. Even gold (Au), the most corrosion-resistant metal, has poor tissue compatibility. Preosteoblasts on Ti and zirconium (Zr) differentiate and calcify more rapidly than those on Au [52]. In addition, the electric plating of platinum (Pt) onto Ti increases corrosion resistance but decreases bone formation because a property of Ti is shielded, thereby preventing bone formation [53]. These results indicate that compatibility with hard tissues is not solely determined by corrosion resistance. In other words, corrosion resistance is a necessary but insufficient condition for biocompatibility; there are other contributing factors.

## 5. The passive film on Ti

### 5.1. Composition and chemical state

**Figure 6.**
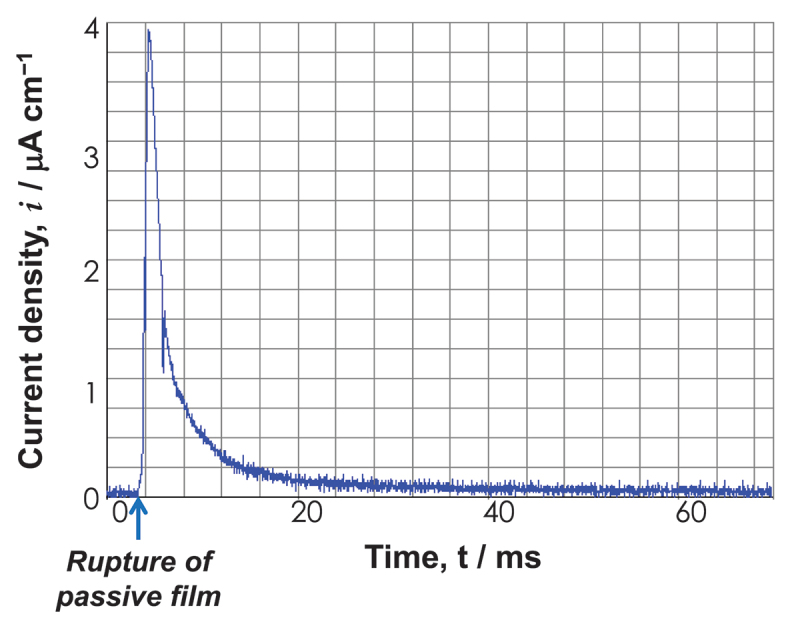
Time transient of current density of Ti after rupturing the passive film by abrasion in Hanks’ solution at 1 V vs. SCE. Positive current is generated both by ion dissolution and the formation of the passive film.

Except in environments of reduction, the corrosion process always results in the formation of a reaction film on metallic materials. Passive film is one such reaction film, and its importance for corrosion protection is especially noteworthy. When solubility is extremely low and pores are absent, film adhesion will be strong to the substrate. The film then becomes a passive or corrosion-resistant film. A passive film has a few nanometers of thickness and is transparent. Passive film readily becomes amorphous as a result of the incredibly rapid rate at which it is formed [54,55]. For instance, a film was generated on a Ti metal substrate in approximately 1/100 s. Figure 6 depicts the transient current density following the rupture of the passive film. In 30 ms, the current density approaches zero, indicating that the passive film is reconstructed immediately. Since amorphous films contain few grain boundaries and structural defects, they are resistant to corrosion. However, crystallization decreases corrosion resistance. Thankfully, passive films contain water molecules that promote and preserve amorphousness.

Metallic Ti naturally form the surface oxide film on itself according to the following equations. In acidic solution, the anodic reactions are: Ti + 2 H_2_O → Ti(OH)_2_ + 2 H^+^ +2e^–^ (oxidation to divalence); Ti(OH)_2_ → TiO + H_2_O (dehydration); TiO + H_2_O → TiOOH + H^+^ + e^–^ (oxidation to trivalence); 2TiOOH → Ti_2_O_3_ + H_2_O (dehydration); Ti_2_O_3_ +3 H_2_O → 2TiO(OH)_2_ +2 H^+^ + 2e^–^ (oxidation to tetravalence); 2TiO(OH)_2_ → TiO_2_  + 2 H_2_O (dehydration). On the other hand, in neutral and basic solutions, the anodic reactions are: Ti + 2OH^–^ → Ti(OH)_2_ + 2e^–^ (oxidation to divalence); Ti(OH)_2_ + OH^–^ → TiOOH^–^ + H_2_O (dehydration); TiOOH^–^ + H_2_O → TiOOH + e^–^ (oxidation to trivalence); 2TiOOH → Ti_2_O_3_ + H_2_O (dehydration); Ti_2_O_3_ + 4OH^–^ → 2TiO(OH)_2_ + H_2_O +2e^–^ (oxidation to tetravalence); 2TiO(OH)_2_ → TiO_2_ +2 H_2_O (dehydration). Since a considerable portion of oxidized Ti stays in Ti^2+^ and Ti^3+^ in the surface film, the oxidation process may proceed to the end just at the uppermost part of the surface film.

**Figure 7.**
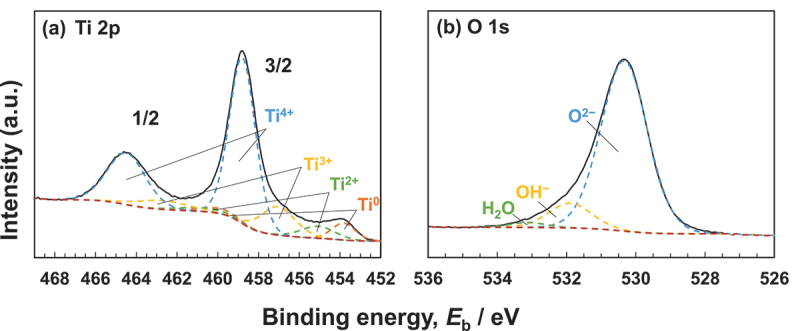
Ti 2p (a) and O 1s (b) electron energy region spectra obtained from Ti immersed in pure water for 1 d and their de-convolutions into component peaks [56].

**Figure 8.**
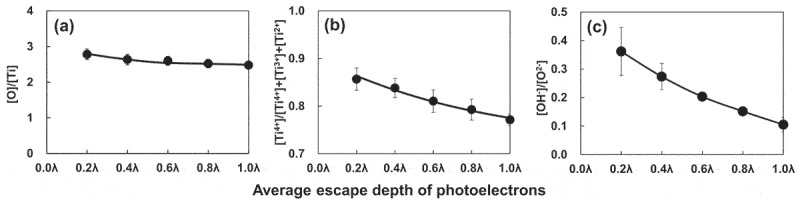
The ratios of [O]/[Ti] (a), [Ti^4+^]/([Ti^4+^]+[Ti^3+^]+[Ti^2+^]) (b), and [OH^‒^]/[O^2^^‒^] (c), plotted against the average escape depth of photoelectrons (*n* = 3) [56]. The angle-resolved technique for XPS was applied to Ti at the photoelectron take-off angles of 12°, 24°, 37°, 53°, and 90°, corresponding to the detection depths of 0.2λ, 0.4λ, 0.6λ, 0.8λ, and 1.0λ, where λ was the photoelectrons’ effective mean free path. The effective escape depth was estimated as λ times the sine of the take-off angle. The take-off angle was defined as the angle between the direction of the photoelectron path to the electron spectrometer and the specimen surface.

Consequently, when Ti is characterized using X-ray photoelectron spectroscopy (×PS), the Ti 2p spectrum exhibited four doublets according to valence: the metallic state of Ti^0^ and the oxide states of Ti^2+^, Ti^3+^, and Ti^4+^, as depicted in Figure 7(a) based on previously published data [56,57]. The decomposition spectrum reveals the presence of Ti^2+^ oxide within the surface oxide layer; however, Ti^2+^ formation is thermodynamically inferior to Ti^3+^ formation at the surface [58–60]. As depicted in Figure 7(b), the spectrum of the O 1s region contained three peaks originating from O^2–^, hydroxide or hydroxyl groups, OH^–^, and hydrate or adsorbed water, H_2_O [61]. Concerning the average effective escape depth of photoelectrons as determined by angle-resolved XPS measurements, λ was the average mean free path of Ti 2p and O 1s photoelectrons, and the effective escape depth was estimated as λ times than the sine of the take-off angle [56,62]; was the average mean free path of Ti 2p and O 1s photoelectrons; Figure 8(a) depicts the ratio of the relative oxygen concentration to that of Ti, [O]/[Ti], versus the average photoelectron escape depth. Oxygen was more abundant in the outer layer of the passive film, while Ti was more abundant in the inner layer. [Ti^4+^]/([Ti^4+^]+[Ti^3+^]+[Ti^2+^]), obtained using the angle-resolved technique, is depicted in Figure 8(b) as the proportion of the integrated intensity of the peak attributed to Ti^4+^ relative to all of its oxide states. At small take-off angles, the percentage of Ti^4+^ was high, indicating that Ti^4+^ was distributed more in the passive film’s outer layer than in its inner layer. In addition, the depth profiles of the [OH^–^]/[O^2–^] ratios are depicted in Figure 8(c), which reveals that OH^–^ was more abundant in the passive film’s outer layer. Consistent with previous research, it is evident that the passive film on Ti consists primarily of an extremely thin TiO_2_ film with trace amounts of Ti_2_O_3_ and TiO, as well as water and hydroxyl groups [55,62,63]. This process of passive film formation has been covered elsewhere [59]. The topmost surface (∼5.0 nm) reveals that the ratio of [TiO_2_]/[Ti_2_O_3_] is consistent with that of passivation/dissolution of electrochemical activity, and that both the structures of passivation, dissolution are distorted due to the appearance of two different sites of Ti–O and Ti–Ti, with bound water in the topmost surface playing a crucial role in structural disorder [64]. It has been determined that the composition, structure, and chemical state of the passive film are distinct from those of crystalline TiO_2_ ceramics. Therefore, the adsorption kinetics of calcium and phosphate ions differ between passive films on Ti and TiO_2_ ceramics [65].

### 5.2. Property as *n*-type semiconductor

**Figure 9.**
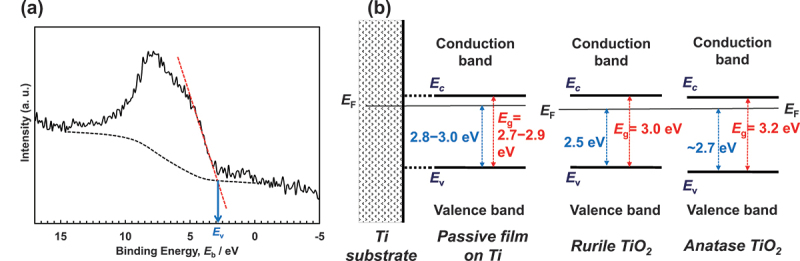
(a) Valence band region spectra of Ti after polarization at 0 V in Hanks for 1 h and the determination of the maximum energy of the valence band, *E*_v_ [66] . (b) Relationship among *E*_g_, *E*_v_, and *E*_F_ in the band structures of the passive film on Ti, rutile TiO_2_, and anatase TiO_2_.

As is common knowledge, TiO_2_ ceramics function as *n*-type semiconductors. How does the passive film on Ti behave as a semiconductor? As shown in Figure 9, the maximum energy of the balance band, *E*_v_, versus Fermi energy, *E*_F_, is determined by linearly extrapolating the peak to the baseline [67] (A). The *E*_v_ value of anatase is approximately 0.2 eV greater than that of rutile [66]. In the case of the passive film on Ti, the *E*_v_ are 2.8–2.9 eV in Hanks’ solution and 2.8–3.0 eV in saline [68], while that in the polished Ti without polarization is 2.8–2.9 eV; this is a higher value than that for rutile, which was 2.5 eV [67]. The observed *E*_v_ value of the as-deposited TiO_2_ film was 1.86 eV [67]. Therefore, the *E*_v_ value for the passive film on Ti was greater than the *E*_v_ value for ceramics composed of TiO_2_. Figure 9 depicts the difference in *E*_v_ versus *E*_F_ between the passive film on Ti and TiO_2_ (B). In other words, the energy between the conduction band’s minimum energy, *E*_c_, and the passive film’s *E*_F_ is less than that of TiO_2_. In addition, as will be explained later, the *E*_g_ of the passive film on Ti is between 2.7 and 2.9 eV, which is significantly less than 3 eV, indicating that the property as an *n*-type semiconductor is much stronger in the passive film on Ti than in the TiO_2_ ceramics.

### 5.3. Dissociation of surface hydroxyl groups – surface electric charge

**Figure 10.**
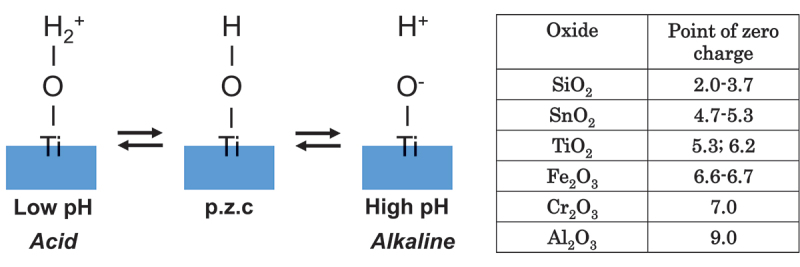
Point of zero charge (p.z.c.) of surface hydroxyl groups on TiO_2_ and their dissociation in aqueous solutions according to pH. The p.z.c. values of metal oxides are listed in the table.

The interface reaction between Ti and living tissue is governed by the passive film property of Ti. This passive film forms hydroxyl groups on their surfaces due to a reaction with atmospheric moisture [69]. In aqueous solutions, such as body fluid, these hydroxyl groups dissociate to form electric charges [69–71]. At a particular pH, the electric charge becomes zero. It is dependent on the pH of the surrounding solution. This pH is defined as the zero-charge point (p.z.c.) (Figure 10). The p.z.c. is specific to each oxide and serves as an indicator of acidic or basic properties (table in Figure 10). In the case of TiO_2_, rutile has a p.z.c. of 5.3 and anatase has a p.z.c. of 6.2 [71]; therefore, TiO_2_ demonstrates neither an acidic nor a basic property, but rather an almost neutral property. The surface concentration of hydroxyl groups on TiO_2_ is relatively high 4.9–12.5 nm^−2^ [70,72]. This concentration or wettability increases when immersed in an aqueous solution. This high concentration promotes the adsorption of proteins, such as integrin and cytokine.

### 5.4. Dielectric constant – electrostatic force

Since proteins are electrically charged objects, adsorption to a metal surface aggravates the conformation of proteins. The electrostatic force exerted by proteins on a metal surface is determined by the relative permittivity of the passive film; the greater the relative permittivity, the lower the electrostatic force. The relative permittivity of TiO_2_ is 82.1, which is significantly greater than that of other oxides and comparable to that of water (80.0) [73]. Consequently, the conformational change of protein adsorbed on TiO_2_ may be minimal. On Ti, the fibrinogen adsorption layer is thicker, but the adsorption amount is less than on Au in aqueous solution [74]. Because Ti is covered by TiO_2_ and Au is exposed without surface oxide, the electrostatic force on Ti is small compared to Au. On Ti, the conformational change of proteins is smaller than on Au. Proteins adsorbed on Ti are more natural.

## 6. Calcium phosphate formation on Ti

**Figure 11.**
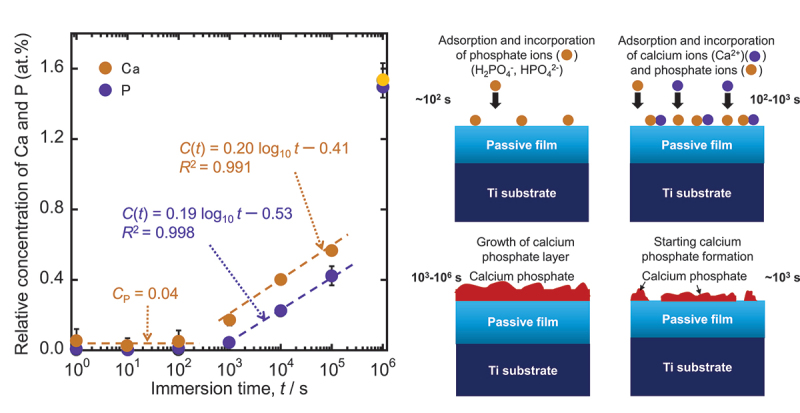
Change in the relative concentrations of calcium and phosphorus in the surface layers of Ti immersed in Hanks’ solution (*n*=3) and illustration of the formation process of calcium phosphate on Ti in Hanks’ solution [63].

Maintaining corrosion resistance, the passive film is macroscopically stable. From a microscopic standpoint, a passive film generally maintains a continuous process of partial dissolution and re-precipitation in the electrolyte [55]. Consequently, the composition and chemical state are affected by the environment. In this way, the surface composition of the passive film is constantly changing in response to its surroundings. Ti and Ti alloys readily form calcium phosphates and sulfite and sulfide in biological environments, particularly under cell culture [75–80]. Recent research [63] has elucidated the initial formation kinetics of calcium phosphate on Ti. First, phosphate ions were incorporated, then calcium ions were incorporated to form calcium phosphate on Ti. Figure 11 demonstrates that calcium and phosphate were incorporated by direct reaction between the Ti substrate and calcium and phosphate ions as calcium and phosphate concentrations increased as immersion time increased from 10^3^ s to 10^5^ s. Additionally, calcium and phosphorus are found at the interface between Ti and bone tissue [81–83]. Zr does not form calcium phosphate against Ti, but rather zirconium phosphate. The passive film on Ti is not fully oxidized and is relatively reactive, whereas the passive film on Zr is more stable and protective than that on Ti [84]. The following section clearly explains these phenomena from the perspective of the *E*_g_. Niobium (Nb) and tantalum (Ta) exhibit properties intermediate to those of Ti and Zr [85]. Electrochemical impedance and photoelectrochemical measurements have characterized the direct interaction of calcium and phosphate ions with the passive film of Ti-6Al-4 V alloy in physiological solutions [86], and calcium phosphate formation on Ti-6Al-4 V alloy is dependent on defects in the passive film [87]. The ability of Ti to form calcium phosphate is therefore one of the reasons for its superior compatibility with hard tissues.

## 7. Band gap energy

### 7.1. TiO_2_ and CaTiO_3_ ceramics

**Figure 12.**
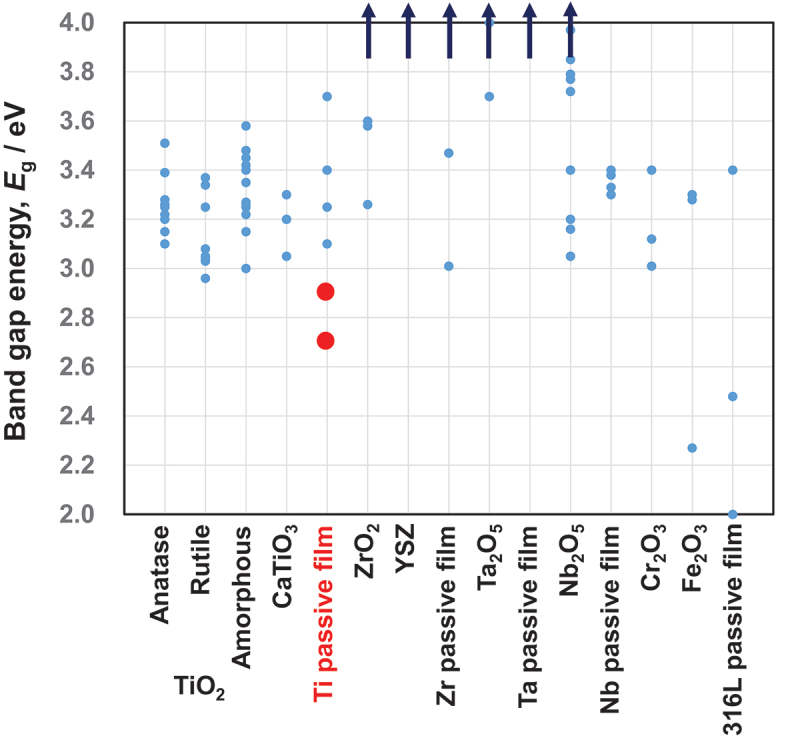
Band gap energies of various oxides and passive films on metals. This figure was originally drawn based on band gap energy data in published papers. The band gap energy of the passive film on Ti in simulated bioliquids (red circles) is relatively low, which may contribute to the reactivity of Ti.

The optical absorption edge is typically used to evaluate the *E*_g_ between the valence and conduction bands in crystalline TiO_2_ ceramics. It is well known that *E*_g_ determines the reactivity of TiO_2_ ceramics, and numerous efforts have been made to reduce *E*_g_ to improve their photocatalyst performance [88]. Therefore, the *E*_g_s of TiO_2_ anatase and rutile are intensively researched for photocatalyst applications. Figure 12 presents a summary of the published data on TiO_2_ ceramics. The *E*_g_ values vary depending on the specimen preparation, which affects oxygen defects and surface morphology.

In the case of anatase, 3.20 ± 0.02 eV for anatase thin film on mica and 3.1–3.2 eV for P25 high-purity fine particle [89], 3.22 eV for pure anatase particle [90], 3.28 eV for anatase and 3.22 eV for anatase high-purity fine particle [91], 3.39 eV for multi-crystal anatase film, 3.51 eV for epitaxial anatase film, and 3.20 eV for bulk anatase [92], 3.15 eV for (001) facet of anatase [93], 3.2 eV for anatase experimented and 3.25 ± 0.12 eV for anatase calculated [94], and 3.26 eV for anatase and 3.20 eV for P25 nanoparticle [95].

In the case of rutile, 3.08 ± 0.02 eV for petaloid rutile [89], 2.96 eV for a pure rutile particle [90], 3.34 eV for multi-crystal rutile, 3.37 eV for an epitaxial rutile film, 3.03 eV for bulk rutile [92], 3.04 eV for the (100) facet of rutile [93], 3.05 eV for rutile experimented and 3.25 ± 0.12 eV for rutile calculated [92], and 3.05 eV for rutile [95].

In the case of brukite, 3.15–3.25 eV was experimentally determined and 3.25 eV is calculated [94].

In the case of amorphous and unidentified TiO_2_ crystals, 3.15–3.25 eV for porous TiO_2_ and 3.22–3.26 eV for nanometer-sized TiO_2_ [91], 3.48 eV for stoichiometric TiO_2_ and 3.35 eV for non-stoichiometric TiO_2_ [96], 3.42–3.45 eV for non-stoichiometric TiO_X_ [97], 3.15–3.27 eV for TiO_2_ nanoparticle fabricated by a sol-gel process [98], and 3.26 eV for TiO_2_ fabricated by a sol-gel process [95].

In the case of calcium titanate, 3.3 eV was measured for CaTiO_3_ and 3.05–3.2 eV is calculated; Ca_3_Ti_2_O_7_ and Ca_4_Ti_3_O_10_ have identical values [94].

### 7.2. The passive film on Ti

Due to its non-stoichiometric composition, the passive film on Ti already contains oxygen defects. Consequently, the difference in the surface properties of passive films on Ti and TiO_2_ ceramics is likely due to the difference in their *E*_g_s. The *E*_g_ of passive films on Ti after anodic oxidation and thermal oxidation has been studied by the photoelectrochemical response in borate buffer solution, artificial sea water, and sulfuric acid [99–101], as conventional techniques for oxide ceramics, such as ultraviolet absorption, cannot be used for thin passive films on Ti. Nevertheless, the *E*_g_s of the aforementioned study are 3.25 ± 0.05 eV for the passive film on Ti anodically polarized in H_2_SO_4_ [99,100], 3.4–3.7 eV for the passive film on Ti anodically polarized in sea water, and 3.1 eV in buffered solution [101]. Moreover, 3.4 and 3.7 eV after 400°C thermal oxidation [101]. However, the oxidation in these studies is excessive, and the passive films are converted to stable TiO_2_ oxide.

**Figure 13.**
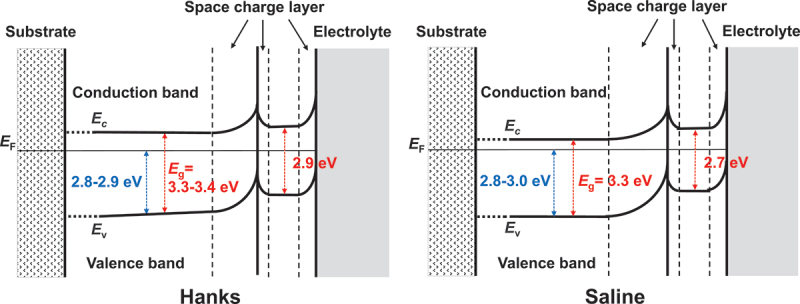
Electronic band structures of passive films formed on Ti in Hanks’ solution and saline [68].

Using the photoelectrochemical response at potentials as close as possible to the open-circuit potential, the *E*_g_ values of passive films formed on Ti in Hanks’ solution and 0.9% NaCl aqueous solution have been evaluated recently [68]. The passive film on Ti behaved like an *n*-type semiconductor with two layers: an inner oxide layer with a high *E*_g_ and an outer hydroxide layer with a low *E*_g_. In Hanks’ solution, the value of *E*_g_ in the innermost layer was between 3.3 and 3.4 eV, whereas it was significantly lower in the surface layer (2.9 eV). *E*_g_ was 3.3 eV in the innermost layer and 2.7 eV in the outermost layer of saline. As shown in Figure 13, the *E*_g_ values of the outermost surfaces of passive films formed on Ti (red circles) were much lower than those of TiO_2_ ceramics [68]. Therefore, the passive film on Ti is more reactive than the ceramic TiO_2_ surface. In addition to the excellent corrosion resistance, this reactivity likely contributes to the excellent biocompatibility of Ti. Calcium phosphate forms regularly on Ti, but not regularly on TiO_2_. The kinetics of calcium phosphate formation on Ti differ from those on TiO_2_ crystalline ceramics [65].

### 7.3. ZrO_2_ and the passive film on Zr

Zr is also a passive metal, whereas the passive film on Zr is a more stoichiometric oxide. ZrO_2_ are well known as bioinert ceramics. Figure 12 also summarizes a portion of the published *E*_g_ data for ZrO_2_ ceramics. The majority exceed the scale of the figure. Theoretical and experimental values are 5.2 eV and 6.5 eV in cubic, 5.2 eV and 6.0 eV in tetragonal, and 5.1 eV and 6.5 eV in monoclinic [102], and 3.26–4.97 eV in cubic, 4.07–4.99 eV in tetragonal, and 3.58–5.34 eV in monoclinic, according to a different study [103]. 5.55 eV for cubic, 6.40 eV for tetragonal, and 5.42 eV for monoclinic are theorized in another study [104]. In monoclinic, the theoretical value is 3.60 eV, while the experimental value is 5.8 eV [105]. Alternatively, experimental values of 5.0–5.8 eV are obtained [106–113]. In the case of ZrO_2_ thin film deposition via sputtering, 4.52 eV is obtained [114]. In thin films of yttria-stabilized ZrO_2_ (16% yttria), 5.62 ± 0.05 eV [115]. In yttria-stabilized cubic ZrO_2_, 4.96 eV [116]. Therefore, ZrO_2_ ceramics have significantly greater *E*_g_ than TiO_2_ ceramics.

In the passive film on Zr, 4.44–4.91 eV in inner oxide layer and 3.01–3.47 eV in hydrated outer oxide layer and 4.3–5.7 eV [117]. 4.42–4.48 eV in Zr-5%(Nb; Mo; Ta; W) alloy [118]. The values are significantly greater than those of Ti.

Zr forms a highly stable and protective passive film, and its reactivity is much lower than that of Ti [84], as described in Section 6 above. *E*_g_ values for ZrO_2_ and passive films on Zr are significantly greater than those for TiO_2_ and passive films on Ti, respectively. This result is supporting that the reactivity of a substance can be determined using *E*_*g*_.

### 7.4. Ta_2_O_5_ and the passive film on Ta; Nb_2_O_5_ and the passive film on Nb

Ta and Nb are also known as passive metals. Ta and Nb are easily oxidized and passivated. Ta and Nb are popularly used for component elements of Ti alloys. Porous Ta is used as a bone defect filler and bone-contacting component in artificial joints. Additionally, Ta is utilized for skull implants and X-ray image markers for stents. Ta is ductile and produces excellent X-ray images due to the heavy metal [34]. Predominant compositions of the passive films are Ta_2_O_5_ and Nb_2_O_5_, respectively [119]. As passive films, Ta_2_O_5_ and Nb_2_O_5_ are highly stable and resistant to corrosion in a biological environment. Ta_2_O_5_ has potential applications in the electronic and catalytic industries. Nb_2_O_5_ is used for catalysts and the production of optical glasses and lithium niobate for solar panel.

As depicted in Figure 12, the *E*_g_s were 4.1 eV [120] and approximately 4 eV [121] for Ta_2_O_5_, 3.7 eV in the γ phase theory [122], and 4.1 eV for the passive film on Ta [123].

*E*_g_s of Nb_2_O_5_ anodic oxidation film on Nb are 3.4 eV [121], 3.33–3.38 eV [124], 3.3 eV [125], 3.4 eV [126], and 3.77–3.85 eV [127]. The *E*_g_s for Nb_2_O_5_ nanoparticles are 3.05 eV nanoparticle [128], 3.72 eV for Nb_2_O_5_ nanotubes and 3.97 eV for Nb_2_O_5_ nanorods [129], and 3.16–4.19 eV for vapor-deposited Nb_2_O_5_ thin films [130]. A review article states that the *E*_g_s of Nb_2_O_5_ are between 3.2 and 5 eV [131].

According to the preceding data, the *E*_g_s of Ta_2_O_5_ and Nb_2_O_5_ are significantly greater than those of TiO_2_. Therefore, the *E*_g_s of the passive film on Ta and Nb are larger than that on Ti: The *E*_g_ of the passive film on Ti is lowest.

### 7.5. *Cr*_2_ *O_3_,Fe_2_O_3_, and the passive film on stainless steels*

Cr_2_O_3_ is the predominant component of passive films on stainless steels and Co–Cr alloys, while Fe_2_O_3_ is sometimes present in passive films on stainless steels. As depicted in Figure 12, the E_g_ of Cr_2_O_3_ nanoparticles varies depending on the firing temperature [132]. The E_g_ of Cr_2_O_3_ has been calculated to be 3.4 eV [133]. The experimental value for Cr_2_O_3_ is 3.4 eV, whereas the theoretical value for Fe_2_O_3_ is 3.28–3.3 eV and the experimental value is 2.27 eV [134]. Therefore, the E_g_ of Cr_2_O_3_ is comparable to that of TiO_2_ and that of Fe_2_O_3_ is lower than that of TiO_2_. The E_g_s of the passive film on austenitic stainless steel type 316 L range between 2.00 and 3.40 eV [94] and 1.95 ± 0.5 eV [135]. In addition, the outer Cr hydroxide layers of the passive film on Fe-Cr alloy have an E_g_ of 2.4 eV [136–140]. Consequently, the E_g_s of the passive film on stainless steels is significantly lower than that on Ti. However, remember that the corrosion resistance of stainless steels is significantly lower than that of Ti.

### 7.6. Relationship between band gap energy and reactivity

According to the review above, the E_g_s of other oxides on passive metals are significantly larger than that of Ti. Therefore, Ti may exhibit optimal reactivity among passive metals, particularly in comparison to Zr. The optimal balance between high corrosion resistance and appropriate reactivity of Ti as a result of the passive oxide film is one of the most important reasons for the excellent biocompatibility of Ti among metals.

## 8. Conclusions

According to the above review and discussion of previous papers, it is possible to explain the excellent biocompatibility of Ti through the following considerations.

- The excellent corrosion resistance of Ti compared to other metals due to a macroscopically strong passive film.
- Ti ions are stabilized immediately to prevent toxicity if released into body fluids.
- Positive and negative charges are well-balanced due to the dissociation of surface hydroxyl groups on the passive film.
- Low electrostatic force of the passive film inducing a natural adsorption of proteins retaining their natural conformation.
- Excellent performance as an *n*-type semiconductor.
- Lower bandgap energy of the passive film on Ti produces optimal reactivity.
- As a result of this reaction, calcium phosphate is naturally formed.

It should be mentioned again that the optimal balance between high corrosion resistance and appropriate reactivity of Ti as a result of the passive oxide film is the most prevalent solution for excellent biocompatibility of Ti. The combination of these properties and the resulting biological response is essential to the elucidation of the biocompatibility of materials as a future spectacular subject. In future, this topic will be essential to better understand the interface phenomena between materials and host bodies using materials informatics (MI) and materials digital transformation (Material DX), because all biological and tissue reactions start from an electronic transfer of the surface.
